# Safety and tolerability of Ashwagandha (*Withania somnifera*) root extract in healthy adults: a prospective, non-comparative study

**DOI:** 10.3389/fnut.2026.1823678

**Published:** 2026-05-21

**Authors:** Navya Movva, Jaysing Salve, Khokan Debnath, Vaishali Thakare, Deepak Langade

**Affiliations:** 1 Department of Pharmacology, D. Y Patil University School of Medicine, Navi Mumbai, India; 2 Department of Internal Medicine, Prakruti Care Hospital, Thane, India; 3 Department of Family Medicine, Prakruti Care Hospital, Thane, India

**Keywords:** adverse events, Ashwagandha, health, high dose safety, SF-36

## Abstract

**Background and aim:**

Ashwagandha, adaptogenic herb traditionally used to enhance resilience to stress and promote overall well-being. Although its safety at conventional doses is well established, evidence regarding the safety of higher doses in healthy adults remains limited. The present study aimed to evaluate the safety and tolerability of Ashwagandha root extract (ARE) administered at a dose of 2000 mg/day in healthy adults over 12 weeks.

**Methods:**

This was a prospective, single-arm, non-comparative clinical study conducted in healthy adults aged 18–65 years. Participants received ARE 500 mg capsules (total daily dose of 2000 mg) orally for 12 weeks. Primary safety outcomes included laboratory evaluations of renal, hepatic, and thyroid parameters. Secondary safety outcomes included clinical assessment of adverse events and changes in quality of life assessed using the SF-36 questionnaire.

**Results:**

Over 12 weeks, minor changes (*p* < 0.05) occurred in serum bilirubin, alkaline phosphatase, creatinine, transaminases, blood urea, and thyroid parameters in PP dataset (*n* = 124) who completed study. All values remained within reference ranges and were clinically insignificant, with most values slightly reduced except a small increase in alkaline phosphatase. No serious or clinically significant changes were noted. In ITT dataset (*n* = 145) most participants (85.5%) experienced no adverse events, with mild nausea being the most common at 5.5%. Quality of life (SF-36 scores) demonstrated improvement (*p* < 0.05) after 12 weeks in the SF-36 domains of physical functioning, role limitations due to physical health, social functioning, and pain.

**Conclusion:**

High-dose Ashwagandha root extract (2000 mg/day) was well tolerated and demonstrated a favourable safety profile in healthy adults over 12 weeks.

**Trial registration:**

https://ctri.nic.in/Clinicaltrials/pmaindet2.php?EncHid=ODE3NTk=&Enc=&userName=, Identifier (CTRI/2023/04/051607).

## 1 Introduction

The term Ashwagandha originates from the Sanskrit words “*ashwa*” (horse) and *“gandha”* (fragrance), referring both to the distinctive smell of the root and the traditional belief that its use promotes strength and vitality similar to that of a horse (1). *Withania somnifera* (L.) Dunal is regarded as an adaptogenic plant and has a long history of use in Ayurvedic medicine for maintaining physiological balance, improving stress resistance, and supporting physical and mental health (2–4).

Among the various parts of the plant, the root is the most utilized and extensively studied. Although Ashwagandha is indigenous to India, it is now cultivated in several regions worldwide, including the Mediterranean area, the Himalayan belt, Africa, the Canary Islands, the Cape of Good Hope, and Australia (5–7). Experimental studies have reported antioxidant, immunomodulatory, and anti-inflammatory effects, supporting its traditional classification as a rejuvenating herb (8).

Studies on Ashwagandha root extract (ARE) have identified multiple bioactive constituents, such as withanolides, alkaloids, and steroidal lactones, which are believed to contribute to its pharmacological and therapeutic properties (9–11). A growing body of clinical research has evaluated standardized ARE in human subjects. These studies have demonstrated benefits in stress reduction, sleep quality, cognitive performance, and physical endurance (12–21). Importantly, available clinical data also indicate that ARE is generally well tolerated. At commonly studied doses, particularly around 600 mg per day, adverse events have been infrequent and typically mild and transient, most often involving gastrointestinal discomfort, headache, or mild drowsiness (12–16, 22–25). Serious treatment-related adverse effects and clinically relevant abnormalities in laboratory parameters have not been reported in these trials.

Despite its extensive use and favorable safety profile at conventional doses, information on the safety of ARE at higher intake levels remains limited. In traditional practice, Ashwagandha root powder is usually consumed at doses of 3–6 g per day, while standardized root extracts are commonly administered in the range of 300–1,000 mg per day, with consistent evidence demonstrating good tolerability and absence of clinically meaningful adverse effects on hepatic, renal and thyroid parameters (26, 27).

With the increasing availability of concentrated extracts and their wider consumption, it is important to generate robust safety data at higher exposure levels. Accordingly, the present study was undertaken to evaluate the safety of Ashwagandha root extract following repeated oral administration. The assessment focused on laboratory biochemical safety parameters and clinical observations over 12 week administration of ARE. The findings of this study are intended to strengthen the existing safety evidence for ARE and to support its responsible use at higher exposure levels.

## 2 Materials and methods

### 2.1 Study design and setting

This prospective, single-arm study was conducted over a 12-week treatment period as per the regulations and Good Clinical Practice (GCP) guidelines, the New Drugs and Clinical Trials (2019) under The Drugs and Cosmetics Act 1940 (India), and the Ethical Guidelines for Biomedical Research on Human Subjects issued by the Indian Council of Medical Research (ICMR). The study was pre-registered with the Clinical Trials Registry of India (CTRI/2023/04/051607; dt.: 13 April 2023).

The study protocol, including all relevant documents, was reviewed and approved by the Institutional Ethical Committees (IECs) of the respective study sites (Approval No.: DYP/IEC/05/2023, dt.: 27 Feb. 2023). The study was conducted at two sites: D Y Patil Medical College Hospital & Research Center, D Y Patil University School of Medicine, Maharashtra, and Prakruti Care Hospital, Maharashtra, India, between September 2024 and December 2024. Participants were recruited and screened for eligibility according to predefined inclusion and exclusion criteria. Prior to participation, informed written consent was obtained from all participants in their vernacular languages to ensure adequate understanding of the study procedures and voluntary participation.

### 2.2 Eligibility criteria

#### 2.2.1 Inclusion criteria

Healthy men and women aged 18 to 65 years were screened for study eligibility. Participants were enrolled if they had no chronic illness, including diabetes, cardiovascular disease, or any other medical condition that could compromise safety or influence study outcomes, and if they had no intention of initiating any alternative or complementary treatment during the study period. Individuals who provided written informed consent and were willing to adhere to all study-related procedures and protocol requirements were included in the study.

#### 2.2.2 Exclusion criteria

Participants were excluded if they had a history of alcohol or smoking abuse, hypersensitivity to Ashwagandha, or were taking nutritional supplements, steroids, or medication. Those with clinical abnormalities, drug abuse history, or concurrent participation in other clinical trials, or who have participated in one during the preceding 3 months, were excluded. Participants who utilized antihypertensive medicine, beta-blockers, inhaled beta-agonists, hormonal contraceptives, or had a history of corticosteroid usage during the past 3 months were eliminated. Participants who had received psychotropic medication in the past 8 weeks, as well as those diagnosed with heart disease, diabetes, stroke, neurological disorders, or depression, and individuals with clinically significant acute, unstable hepatic, renal, cardiovascular, or respiratory diseases, were excluded from the study. Patients with depressive episodes, panic disorder, social phobia, obsessive-compulsive disorder, alcohol dependence, schizophrenia, mania, and post-traumatic stress disorder were excluded. Individuals with a meditation practice of 3 months or more, as well as pregnant and nursing mothers, were excluded.

To ensure participant safety and to minimize potential confounding effects on the study outcomes, strict inclusion and exclusion criteria were applied. Medications and supplements known to influence stress physiology, neuroendocrine function, cardiovascular parameters, metabolic status, or psychological outcomes were not permitted during the study. A uniform washout period of 8 weeks was applied to all psychotropic medications to allow adequate clearance and stabilization of neurobiological effects prior to baseline assessment.

*Participant withdrawal criteria*: participants were withdrawn from the study in the case of voluntary withdrawal of consent, non-compliance with the study protocol, occurrence of serious adverse events requiring discontinuation, or at the discretion of the investigator. In cases of withdrawal, the reason for discontinuation and any adverse events were documented, and all available assessments up to the time of withdrawal were recorded. Follow-up evaluations were attempted, particularly for treatment-related adverse events, and a final evaluation was completed where feasible.

### 2.3 Interventions

Participants received 500 mg capsules of Ashwagandha root extract (ARE; KSM-66^®^ Ashwagandha, manufactured by Ixoreal Biomed Inc., Los Angeles, California, USA). Participants were instructed to take two capsules orally twice daily, once after breakfast and once after dinner, with a glass of water, for a duration of 12 weeks. This dosing regimen corresponded to a total daily dose of 2000 mg of ARE.

The investigational product, KSM-66 Ashwagandha^®^ capsules is a root-only extract manufactured using a green chemistry method (aqueous-based extraction process) that is devoid of alcohol or chemical solvents. The extract contains >5% withanolides, quantified by high-performance liquid chromatography (HPLC), with a drug-to-extract ratio of 12:1. The product is a light yellowish powder with a neutral taste.

### 2.4 Study outcomes

The primary outcome of the study was safety of ARE assessed by changes in the laboratory parameters from baseline to week 12, including renal, liver function, and thyroid function from baseline to week 12. The secondary outcome assessed the number and proportion of treatment-emergent adverse events (TEAEs) and treatment-emergent serious adverse events (TESAEs) during the 12-week treatment period. Additionally, changes in the SF-36 total score were evaluated to measure quality of life (QoL) improvements.

#### 2.4.1 Study assessments

At the initial visit, prospective patients were screened for eligibility based on the study requirements, medical history, and clinical examination. After screening, demographic characteristics such as date of birth, age, gender, height, weight, and BMI were recorded at baseline. Additional details, including annual income, educational qualifications, and marital status, were also documented.

Data were collected at screening/enrolment (Baseline, Day −3 to Day 0), Week 4 (±4 days), Week 8 (±4 days), and Week 12 (±4 days) according to a predefined study schedule. No protocol amendments were made following ethics approval.

##### 2.4.1.1 Laboratory procedures

Laboratory investigations, including renal parameters (serum creatinine and blood urea nitrogen), liver function parameters (serum alanine transaminase, aspartate transaminase, alkaline phosphatase, and bilirubin), and thyroid function tests (serum thyroid-stimulating hormone, triiodothyronine, and thyroxine), were assessed throughout all the visits. Blood samples were collected using standard venipuncture techniques following routine clinical laboratory practices. All analyses were performed using standardized, validated methods at a certified diagnostic laboratory in accordance with applicable quality and regulatory standards.

Laboratory investigations were assessed at Visit 1, Screening Visit/ Enrolment Visit/ Baseline Visit, (Day 1), Visit 2 (Week 4), Visit 3 (Week 8), and Visit 4- End of study visit, (Week 12).

### 2.5 Safety assessment

Safety was assessed through continuous monitoring of adverse events (AEs) from the time of informed consent until study completion. AEs were identified based on spontaneous reports by participants and clinical observations by the investigator at scheduled and unscheduled visits and were documented on the adverse event pages of the Case Report Form. An AE was defined as any untoward medical occurrence or clinically significant abnormal laboratory finding representing a change from baseline, irrespective of causal relationship. Severity was graded as mild, moderate, or severe based on impact on daily activities. Causality was assessed by the investigator considering temporal relationship, known product profile, and alternative etiologies and classified as related (possible or probable), suspected, or not related. Serious adverse events (SAEs) were defined according to ICH-GCP criteria and reported to the sponsor and ethics committee within regulatory timelines. Expectedness was assessed with reference to the Ashwagandha 500 mg capsule product information. Recurrent events with similar characteristics were recorded as a single AE with multiple episodes, while distinct events were documented separately and followed until resolution or stabilization.

### 2.6 Short form survey (SF-36)

The SF-36 is a self-reported questionnaire assessing health-related quality of life (QoL) across eight domains. In this study, individual domain scores were assessed and analyzed. Participants completed the 36-item questionnaire at baseline, week 4 and week 12 (28). SF-36 outcomes were analyzed and reported separately for each of the eight domains, as recommended for this instrument, with higher scores indicating better quality of life. The SF-36 was a secondary outcome to explore the efficacy of ARE in high dose administration for 12 weeks.

### 2.7 Sample size

A total of 100 participants were planned for enrolment, considering the incidence of adverse events reported in previous studies on ARE, which ranged from 4% to 35% (29, 30). Based on a 35% incidence rate, a sample size of 63 would provide 80% power to detect this event rate with a null hypothesis value of 20% at an alpha level of 0.05 (95% confidence levels). However, to account for potential data loss, the study enrolled 145 participants.

### 2.8 Statistical methods

All statistical analyses were performed on both the intent-to-treat (ITT) and per-protocol (PP) datasets, as prespecified in the study protocol. The ITT population included all enrolled participants who received at least one dose of the investigational product and had at least one post-baseline assessment, irrespective of study completion status, and was primarily used for safety assessments. The PP population included participants who completed the study in accordance with the protocol without major protocol violations.

The data were analyzed utilizing Stata IC/13 (Stata Corp LLC, USA), a Windows-based statistical software. Measurement data and total scores were summarized as means with standard deviations (SD). Categorical variables were presented as counts and percentages, while ordinal data were summarized using means, medians, quartiles, and SD. Changes from baseline were calculated and expressed as both absolute mean change and percentage change from baseline. Ninety-five percent confidence intervals (95% CIs) were presented where applicable.

Analyses were done using a mixed effect model (repeated-measures analysis of variance) for normal data. Subgroups analyses were done for gender and WHO BMI categories using mixed effects model. All statistical tests were two-sided with a significance level of *α* = 0.05. No data transformations were applied prior to analysis. Outliers were not excluded unless attributable to data entry errors, no formal multiple-comparison adjustments were performed, and missing data were not imputed; analyses were conducted using observed data only.

## 3 Results

A total of 145 healthy participants (88 men and 57 women) were enrolled and comprised the intent-to-treat (ITT) population. Of these, 21 participants were lost to follow-up, resulting in a per-protocol (PP) population of 124 participants who completed the study without major protocol deviations. Of the 21 participants, 5 were lost to follow-up (reasons unknown), 3 withdrew consent at 4 weeks, and 13 had poor compliance. Reasons for poor compliance and withdrawal of consent were not related to study intervention.

The ITT dataset (*n* = 145) included all patients recruited in the study who had taken at least one dose of Investigational Product and had given at least one post-baseline assessment, irrespective of their study completion status, whereas the PP dataset (*n* = 124) included all patients who completed the study as per the protocol without any protocol violation (Table 1).

**Table 1:** Baseline profile, demography and BMI of patients in ITT and PP datasets.

	ITT dataset (*n* = 145)		PP dataset (*n* = 124)	
Gender	No.	%	No.	%
Male	88	60.7%	74	59.7%
Female	57	39.3%	50	40.3%
BMI category (WHO South Asia)	No.	%	No.	%
Underweight (<18.5 kg/m^2^)	2	1.4%	2	1.6%
Normal weight (18.5 to 22.9 kg/m^2^)	53	36.8%	45	36.6%
Overweight (23.0 to 27.4 kg/m^2^)	61	42.4%	52	42.3%
Obese (≥27.5 kg/m^2^)	28	19.4%	24	19.5%

	ITT dataset (*n* = 145)		PP dataset (*n* = 124)	
Demography	Mean	SD	Min.	Max.
Age (yrs.)	39.26	12.98	18	65
SBP (mm Hg)	125.63	10.26	96	152
DBP (mm Hg)	82.87	12.20	58	115
Pulse rate (per min.)	83.23	12.52	52	110
Respiratory rate (per min.)	19.08	5.05	14	78
Body temperature (°F)	96.90	0.57	95.7	98.7
BMI (kg/m^2^)	26.37	4.55	17.36	45.44
Hepatic parameters	Mean	SD	Min.	Max.
Total bilirubin (mg/dL)	0.80	0.38	0.29	4.00
Direct bilirubin (mg/dL)	0.37	0.20	0.02	0.70
Indirect bilirubin (mg/dL)	0.40	0.19	0.09	1.00
Alkaline phosphatase (IU/l)	106.69	44.76	2.40	197.00
AST/SGOT (U/L)	30.85	8.45	12.00	60.00
ALT/SGPT (U/L)	29.37	9.46	1.00	63.00
Renal parameters	Mean	SD	Min.	Max.
Creatinine (mg/dL)	0.94	0.75	0.29	9.50
BUN (mg/dL)	17.01	4.96	0.80	28.00
Thyroid profile	Mean	SD	Min.	Max.
TSH (uIU/ml)	1.00	0.89	0.20	5.00
T3 (ng/ml)	1.28	0.48	0.61	2.03
T4 (ug/dl)	9.81	6.08	2.90	23.00
QoL SF-36 domain	Mean	SD	Min.	Max.
Physical functioning	53.34	27.60	0.00	100.00
Role limitation due to physical health	44.25	37.89	0.00	100.00
Role limitation due to emotional problem	42.52	39.97	0.00	100.00
Energy\fatigue	50.59	13.04	25.00	85.00
Emotional Well-being	54.51	12.76	20.00	88.00
Social functioning	59.59	19.57	15.00	100.00
Pain	53.21	22.62	10.00	100.00
General health	57.57	15.27	25.00	100.00
SF-36 total score	75.09	13.47	74.65	13.72

No., number of patients; BMI, body mass index; SBP, systolic blood pressure; DBP, diastolic blood pressure; SD, standard deviation; QoL, quality of life; ALT/SGPT, alanine aminotransferase; AST/SGOT, aspartate aminotransferase; BUN, Blood Urea Nitrogen; TSH, thyroid-stimulating hormone; T3, triiodothyronine; T4, thyroxine.

### 3.1 Primary outcome

#### 3.1.1 Laboratory investigations

Table 2 summarizes the baseline and follow-up laboratory values for various laboratory parameters up to week 12.

**Table 2:** Baseline and change from baseline in laboratory parameters in PP dataset (*n* = 124).

	References range	Baseline	Week 12	Change from baseline	Paired *t*-test
Hepatic parameters		Mean (SD)	Mean (SD)	Mean (SD)	*p*
Total Bilirubin (mg/dL)	0.1–1.2	0.80 (0.40)	0.73 (0.26)	−0.07 (0.43)	0.026
Direct Bilirubin (mg/dL)	0.0–0.4	0.38 (0.20)	0.28 (0.16)	−0.10 (0.22)	0.002
Indirect Bilirubin (mg/dL)	0.2–0.8	0.40 (0.18)	0.37 (0.19)	−0.03 (0.23)	0.025
Alkaline Phosphatase (IU/l)	40–129	103.61 (45.08)	104.42 (41.20)	0.80 (32.67)	<0.0001
AST/SGOT (U/L)	10–40	30.76 (8.34)	28.05 (5.33)	−2.71 (8.31)	<0.0001
ALT/SGPT (U/L)	7–56	29.52 (8.95)	26.70 (5.10)	−2.83 (8.12)	<0.0001
Renal parameters		Mean (SD)	Mean (SD)	Mean (SD)	*p*
Creatinine (mg/dL)	0.6–1.2 (M)	0.95 (0.81)	0.80 (0.25)	−0.15 (0.80)	0.046
	0.5–1.1 (F)				
BUN (mg/dL)	7–20	16.91 (5.04)	16.11 (4.09)	−0.80 (4.99)	<0.0001
Thyroid profile		Mean (SD)	Mean (SD)	Mean (SD)	*p*
TSH (uIU/ml)	0.4–4.0	0.99 (0.84)	0.96 (0.96)	−0.03 (0.69)	<0.0001
T3 (ng/ml)	0.8–2.0	1.26 (0.48)	1.20 (0.43)	−0.06 (0.14)	<0.0001
T4 (ug/dl)	5.0–12.0	9.76 (6.22)	9.58 (6.00)	−0.18 (0.92)	<0.0001

SD, standard deviation; ALT/SGPT, alanine aminotransferase; AST/SGOT, aspartate aminotransferase; TSH, thyroid-stimulating hormone; T3, triiodothyronine; T4, Thyroxine; BUN, blood urea nitrogen; M, male; F, female.

Total bilirubin showed a statistically significant decrease from baseline to Week 12 (mean change −0.07 ± 0.43 mg/dL; *p* = 0.026). Significant reductions were also observed in direct bilirubin (−0.10 ± 0.22 mg/dL; *p* = 0.002) and indirect bilirubin (−0.03 ± 0.23 mg/dL; *p* = 0.025). Alkaline phosphatase demonstrated a small but statistically significant increase (0.80 ± 32.67 IU/L, *p* < 0.0001). Serum aspartate aminotransferase (AST/SGOT) and alanine aminotransferase (ALT/SGPT) levels decreased significantly from baseline (−2.71 ± 8.31 U/L and −2.83 ± 8.12 U/L, respectively; both *p* < 0.0001).

Serum creatinine decreased significantly from baseline to week 12 (−0.15 ± 0.80 mg/dL; *p* = 0.046). Blood urea nitrogen (BUN) also showed a statistically significant reduction (−0.80 ± 4.99 mg/dL; *p* < 0.0001).

Thyroid-stimulating hormone (TSH) showed a small but statistically significant decrease from baseline (−0.03 ± 0.69 μIU/mL; *p* < 0.0001). Statistically significant reductions were also observed in serum triiodothyronine (T3; −0.06 ± 0.14 ng/mL; *p* < 0.0001) and thyroxine (T4; −0.18 ± 0.92 μg/dL; *p* < 0.0001).

All laboratory values remained within normal reference limits for all participants in the study. Although several laboratory parameters showed statistically significant changes (*p* > 0.05) from baseline, all values remained within clinically acceptable reference ranges, and no laboratory abnormalities of clinical significance were observed. No findings required medical intervention or were considered indicative of hepatic, renal, or thyroid dysfunction.

### 3.2 Secondary outcomes

#### 3.2.1 Adverse events and safety assessment in the ITT dataset

No serious adverse events (SAEs) were reported during the study. Among the 145 participants included in the safety (ITT) population, 124 participants (85.5%) reported no adverse events, while 21 participants (14.5%) experienced at least one adverse event (AE).

A total of 19 adverse events were reported during the study. The most frequently reported AE was nausea, occurring in 8 participants (5.5%), followed by abdominal pain in 5 participants (3.4%), drowsiness in 3 participants (2.1%), and dizziness in 3 participants (2.1%). All reported adverse events were mild in severity, transient, and resolved without medical intervention. No participants were withdrawn from the study due to adverse events.

Causality assessment was performed by the investigator in accordance with the study protocol. The reported adverse events were assessed as possibly or probably related to the investigational product. No adverse events were assessed as severe or serious.

In accordance with the protocol, when the same adverse event occurred more than once in a participant with similar characteristics (severity, causality, and outcome), it was recorded as a single adverse event with multiple episodes. Distinct adverse events were documented separately.

Overall, Ashwagandha was well tolerated, with a low incidence of mild adverse events and no serious adverse events, supporting its favorable safety profile in healthy adult participants.

#### 3.2.2 Sf-36 (QOL)

Changes in health-related quality of life assessed using the SF-36 questionnaire in the per-protocol (PP) population (n = 124) are summarized in Table 3. The SF-36 scores demonstrated improvement across all domains from baseline to weeks 4 and 12.

**Table 3:** Baseline and change from baseline of SF-36 QOL score in PP dataset (*n* = 124).

SF-36 domain	Baseline	Week 4	Change From baseline	Week 12	Change From baseline	p*
	Mean (SD)	Mean (SD)	Mean (SD)	Mean (SD)	Mean (SD)	
Physical functioning	53.63 (27.39)	67.81 (20.76)	15.17 (26.57)	83.66 (22.30)	30.03 (34.97)	<0.0001
Role limitation due to physical health	43.28 (38.75)	69.79 (32.39)	28.13 (37.74)	80.04 (31.56)	36.76 (49.21)	0.003
Role limitation due to emotional problem	44.08 (40.45)	64.45 (36.61)	20.84 (49.29)	85.39 (24.58)	42.23 (45.82)	0.315
Energy\fatigue	49.92 (13.19)	55.29 (16.77)	5.25 (20.01)	62.81 (15.78)	12.89 (19.99)	0.947
Emotional well-being	53.71 (12.73)	67.34 (13.02)	13.75 (16.26)	74.60 (14.18)	20.89 (17.03)	0.583
Social functioning	59.48 (19.42)	72.62 (14.46)	13.84 (19.70)	82.96 (18.68)	23.49 (28.43)	<0.0001
Pain	53.08 (22.58)	67.46 (21.69)	15.37 (22.10)	77.22 (23.55)	24.33 (28.20)	0.001
General health	57.39 (15.34)	65.47 (16.29)	8.76 (19.77)	70.77 (17.95)	13.38 (20.80)	0.754

*Repeat measures ANOVA.

Higher scores give better quality of life.

QoL, Quality of life; SD, Standard deviation; SF-36, Short form 36.

Across domains, the magnitude of change was greatest in physical functioning (30.03 ± 34.97), role limitation due to physical health (36.76 ± 49.21), and role limitation due to emotional problems (42.23 ± 45.82), while smaller gains were observed in energy/fatigue (+12.89 ± 19.99) and general health (13.38 ± 20.80). Pain (24.33 ± 28.20) and social functioning (23.49 ± 28.43) showed intermediate improvements.

Thus, statistically significant enhancements were noted in physical functioning (*p* < 0.0001), role limitation due to physical health (*p* = 0.003), social functioning (*p* = <0.0001), and pain (*p* = 0.001) domains. Other domains, including role limitation due to emotional problems, energy/fatigue, emotional well-being, and general health, exhibited improvements that were not statistically significant (*p* > 0.05).

**Figure 1:**
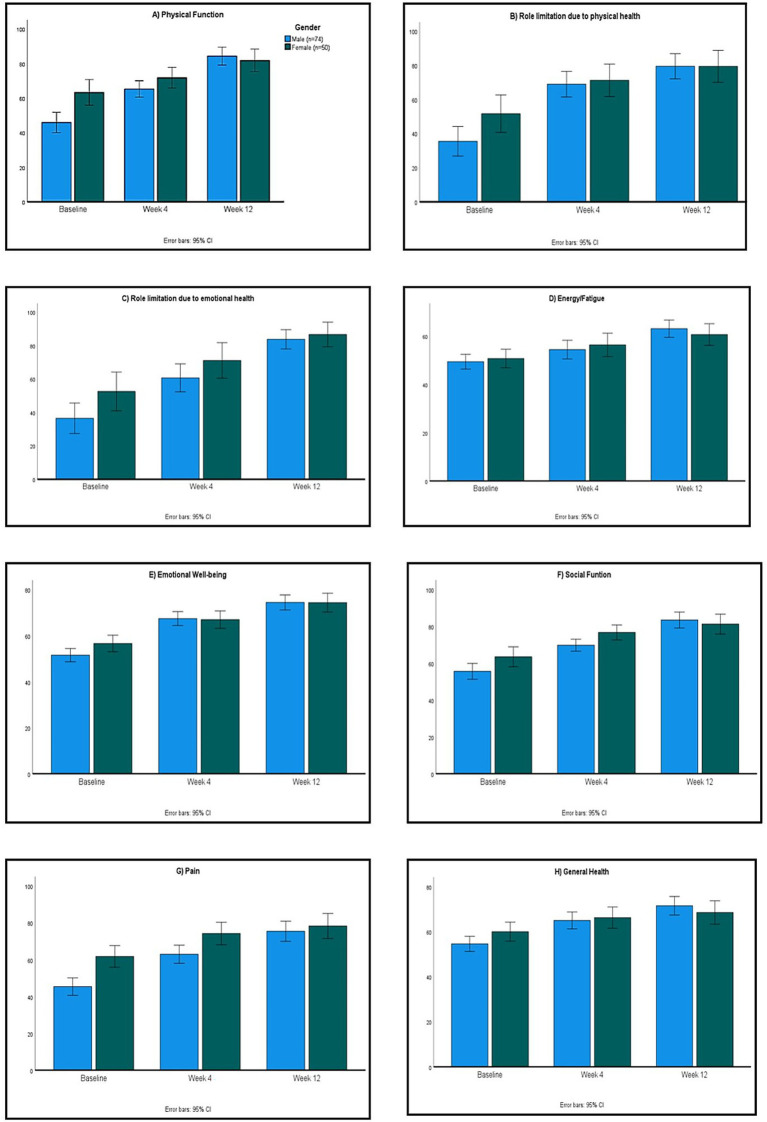
SF-36 domain scores for quality of life (QoL) in males and females in the per-protocol (PP) dataset. Domains include physical functioning **(A)**, role limitations due to physical health **(B)**, role limitations due to emotional problems **(C)**, energy/fatigue **(D)**, emotional well-being **(E)**, social functioning **(F)**, bodily pain **(G)**, and general health **(H)**. (*p* < 0.01 for comparison of scores between males versus females).

These improvements were seen as early as week 4 following ARE administration. These findings indicate a positive impact of ARE on QoL over the 12-week study period. Overall, women had greater improvement (*p* < 0.01) in QoL compared to men (Figure 1).

**Figure 2:**
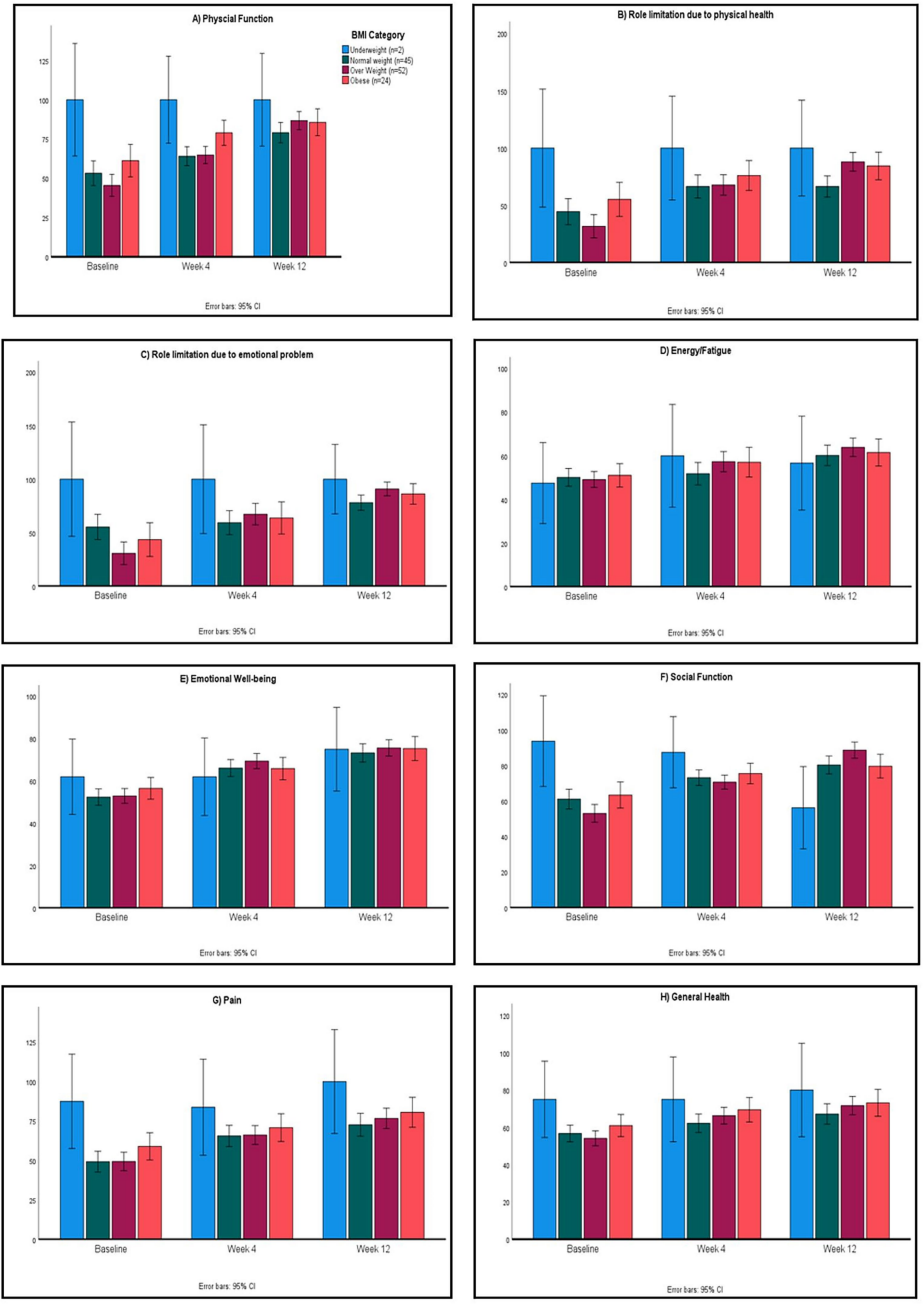
SF-36 domain scores for quality of life (QoL) across body mass index (BMI) categories in the per-protocol (PP) dataset. Domains include physical functioning **(A)**, role limitations due to physical health **(B)**, role limitations due to emotional problems **(C)**, energy/fatigue **(D)**, emotional well-being **(E)**, social functioning **(F)**, bodily pain **(G)**, and general health **(H)**. (*p* > 0.05 for comparison of scores between BMI categories).

Subgroup analyses revealed higher enhancements in SF-36 domain scores in women relative to men (*p* < 0.01; Figure 1). Participants classified as obese exhibited greater increases in SF-36 scores compared to those with normal or underweight body mass index; however, these differences did not reach statistical significance (*p* > 0.05; Figure 2).

## 4 Discussion

This single-arm, non-comparative, prospective clinical investigation was designed to evaluate the safety and tolerability of a high dose of Ashwagandha (*Withania somnifera*) root extract (ARE) in healthy adults. To the best of our knowledge, this is the first clinical study to systematically report safety outcomes following administration of ARE at 2000 mg/day, a dose substantially higher than those evaluated in most previously published trials. Given the increasing use of Ashwagandha in health and wellness settings, systematic evaluation of its safety at higher extract doses is needed. While Ashwagandha has a long history of traditional use, modern clinical evidence has largely been limited to lower doses and shorter exposure durations.

Several randomized controlled trials and systematic reviews have demonstrated the safety of Ashwagandha at conventional doses (typically 120–600 mg/day) (31, 32). Consistent with this literature, the present study demonstrated a favorable safety profile at a substantially higher dose (2000 mg/day). Over 12 weeks of administration, no clinically meaningful changes were observed in hepatic, renal, or thyroid parameters, and all laboratory values remained within reference limits. A total of 21 participants (14.5%) reported adverse events, the majority of which were mild and transient, with nausea being the most frequently reported event. No serious adverse events or study discontinuations due to safety concerns were observed. The frequency and nature of adverse events are comparable to those reported in prior Ashwagandha studies, where adverse event rates have ranged from approximately 7% to 35%, predominantly involving mild gastrointestinal or sleep-related symptoms (27, 33).

These findings are supported by earlier clinical investigations. Verma et al. (2) reported that ARE administered at 600 mg/day for 8 weeks was well tolerated in healthy volunteers, with no clinically significant changes in hematological, hepatic, renal, or thyroid parameters. Chandrasekhar et al. reported mild discomfort in approximately 30% of participants, while other studies have reported lower adverse event rates ranging from 7.5% to 16.7% (4). Longer-term evidence further supports the safety of ARE. A randomized, double-blind, placebo-controlled study evaluating ARE at 300 mg twice daily for 24 weeks demonstrated comparable adverse event rates between ARE and placebo groups, with no clinically meaningful alterations in laboratory safety parameters (27). Similarly, a prospective multicentre observational study involving 12 months of ARE administration reported only mild, self-limiting adverse events and stable laboratory parameters, indicating the favorable safety profile of ARE during prolonged use (34).

Although the primary objective of the present study was safety, exploratory quality-of-life assessments using the SF-36 questionnaire demonstrated domain-specific improvements over the 12-week study period. Prior evidence regarding quality-of-life outcomes with Ashwagandha is mixed and varies by population and assessment instrument. Some studies have reported improvements in perceived well-being and mental health domains, including trials using the SF-12 questionnaire, while a systematic review and meta-analysis by Cheah et al. (31) found no consistent overall effect of Ashwagandha on quality of life despite improvements in sleep-related outcomes. Given these inconsistencies and the exploratory nature of SF-36 analyses in the present study, quality-of-life findings to be interpreted cautiously and viewed as supportive rather than confirmatory. Importantly, subgroup analyses suggesting greater improvements among women or participants with obesity did not demonstrate statistically significant between-group differences and therefore do not support definitive subgroup effects.

Preclinical studies demonstrating enhanced stress resistance and lifespan extension (35, 36) in model organisms provide mechanistic support for Ashwagandha’s traditional classification as a rejuvenative herb; however, such findings cannot be directly extrapolated to humans and should be interpreted within a translational context.

### 4.1 Limitations and future directions

Several limitations need consideration. The single-arm, non-comparative design limits causal inference and precludes direct comparison with a placebo. The observational nature of the study may also introduce selection and reporting biases. The study provides important initial safety data for high-dose ARE administration. Future research should include larger, randomized, placebo-controlled trials to confirm these findings and to further evaluate the long-term safety of high-dose Ashwagandha across diverse populations, with extended follow-up to assess chronic use beyond 3 months.

## 5 Conclusion

This clinical study found that a daily oral dose of 2000 mg Ashwagandha (*Withania somnifera*) root extract was safe and well tolerated over 12 weeks in healthy adults, with no impact on liver, kidney, or thyroid function. These findings provide novel evidence supporting the high dose Ashwagandha supplementation and establish a foundation for future long term and efficacy studies. Further controlled studies are warranted to assess long-term safety and broader benefits.

## Data Availability

The raw data supporting the conclusions of this article will be made available by the authors, without undue reservation.
